# Association of Fear of COVID-19 and Health Literacy Among the General Public in Saudi Arabia: A Cross-Sectional Assessment

**DOI:** 10.3389/fpubh.2021.740625

**Published:** 2022-01-21

**Authors:** Mahaman L. Moussa, Fatchima Laouali Moussa, Homood A. Alharbi, Tagwa Omer, Hussain Ahmad Sofiany, Tarraji Mohammed Almuzaini, Eissa Salem Alsaady, Sattam Musleh Alrashede

**Affiliations:** ^1^College of Nursing, King Saud University, Riyadh, Saudi Arabia; ^2^College of Nursing, Princess Nourah Bint Abdulrahman University, Riyadh, Saudi Arabia; ^3^College of Nursing, King Saud bin Abdulaziz University for Health Sciences, Jeddah, Saudi Arabia; ^4^Ohud Hospital Ministry of Health, Madina, Saudi Arabia

**Keywords:** COVID-19, fear, health literacy, pandemic, Saudi Arabia

## Abstract

**Objective:**

This study aims to determine the level of fear of coronavirus disease—2019 (COVID-19) among the general public in Saudi Arabia and the association of its scores with their level of health literacy.

**Methods:**

A descriptive cross-sectional study was conducted among the general population in Saudi Arabia from April 2020 to May 2020. A three-part online self-reported survey was used to assess the participants' demographic characteristics, fear of COVID-19 of the participants, and levels of health literacy.

**Results:**

Of the 848 participants, 56.8% were in the age group of 25–34, 45.6% were females, and 57.1% had a bachelor's degree. The total mean score of the fear of COVID-19 scale of the participants was FCoV-19: mean ± SD = 19.60 ± 7.33 and the health literacy was HL index: mean ± SD = 27.57 ± 11.05. There was a significant difference in the scores of fear of COVID-19 scale in terms of age (*F* = 2.442, *p* = 0.050), representing that those aged 45 and above had higher mean fear scores than the younger participants. The analysis revealed that an increased level of fear of COVID-19 was associated with older age (*B* = 1.87; *p* = 0.020), being unemployed (*B* = 0.76; *p* = 0.023), with no formal education (*B* = 0.89; *p* = 0.001), and low level of health literacy (*B* = 0.02; *p* = 0.021).

**Conclusion:**

The study shows an above-average level of fear of COVID-19 of the general public in Saudi Arabia and its association with a low level of functional health literacy. Timely and comprehensive health interventions should be promoted to enhance the level of health literacy and further reduce the level of fear of COVID-19 in the community.

## Introduction

The novel coronavirus disease—2019 (COVID-19) was a virus first identified in Wuhan, China last December 2019 (1). This pneumonia-like disease has caused significant damage globally, which affects more than 227 countries (2). Because of the unprecedented damage to public health, WHO declared COVID-19 as a pandemic. At present, cases of severe acute respiratory syndrome coronavirus 2 (SARS-CoV-2) had reached more than 40 million with 782,456 confirmed deaths worldwide (3). In Saudi Arabia, there are more than 300,000 confirmed cases (3). The COVID-19 pandemic has challenged different leaders to address the sporadic spread of the disease. Different governments around the world have enforced several preventive measures such as flight restriction, lockdowns, and quarantine to contain and prevent transmission (4).

The emergence of COVID-19 with its severity and high rate of transmission created fear, feeling of uncertainty, and anxiety in the public (5–7). Different myths and misinformation have also brought concern that causes a panic of being infected (6, 7). Several countries reported elevated levels of fear of COVID-19 during the first outbreak (8–10). In Canada and the United States identified fears about economic consequences and traumatic stress symptoms were associated with the COVID-19 pandemic (9). Similar findings were conducted in Italy in which four domains of fear during the COVID-19 pandemic were identified using a conceptual analysis; (1) fear for the body, (2) fear for significant others, (3) fear of not knowing, and (4) fear of inaction (10). Fear is a natural emotion and adaptive emotion that serves to mobilize energy to deal with potential threats (11). The fear of COVID-19 is considered one of the vital factors that might produce and leads to elevated levels of anxiety and stress during the pandemic particularly fear of getting infected or infecting their loved ones (12). Reports have shown that those who have a better perception of health information or have a higher degree of health literacy are the least likely to have higher anxiety levels (13, 14).

The concept of health literacy is defined as individuals' cognitive and social skills to access, understand, and apply the health information needed to facilitate decisions concerning healthcare (15, 16). A previous study reported that patients with inadequate health literacy have problems or experiencing difficulties such as understanding health educational materials, reading medication labels, and communicating with their healthcare provider which affects disease management (17). In addition, patients with low health literacy almost experience adverse health outcomes three times (17). Meanwhile, a recent study conducted among medical students highlights that health literacy is essential to respond proactively to COVID 19 such as it helps in preventing over-reaction and carelessness (18). At present, health literacy may play an important role that can assist healthcare policymakers and health system managers in planning health interventions to protect people from COVID-19. Less is known about the level of fear of COVID-19 of the general public in Saudi Arabia as well as their level of health literacy. Thus, gaining a better understanding of the factors associated with health literacy may assist in forming specific public health strategies during the COVID-19 pandemic. This study aims to determine the level of fear of COVID-19 among the general public in Saudi Arabia and the association of its scores with their level of health literacy.

## Methods

### Design and Participants

A descriptive cross-sectional study was conducted among the general population of Saudi Arabia from April 2020 to May 2020. Because of the restricted measures and lockdowns imposed by the government, a convenience sampling was used and the data was collected through an online self-reported survey using Google forms. All participants aged 18 and above were invited to participate in this study. Invitations to take part in the study were shared using a weblink that was distributed through social media platforms (Facebook, Twitter, LinkedIn, and WhatsApp). Furthermore, a snowball technique was used to disseminate the questionnaire as we requested the participants to share the survey link to their family, relatives, and friends. The Institutional Review Board General Directorate of Health Affairs in Madinah (IRB no: 567, H-03-M-084) approved this study, with which the authors are affiliated.

### Measures

The questionnaire consisted of three parts: first, the demographic characteristics of the participants, which included age, gender, marital status, employment status, nationality, educational attainment, and monthly income. Second, the fear of COVID-19 Scale (FCOV-19S) was used to assess the levels of fear of COVID-19 of the participants. FCOV-19S is a 7-items questionnaire that was developed by Ahorsu et al. (19). FCOV-19S is a valid, reliable instrument that was also validated used in countries such as Vietnam, Bangladesh, and Japan (18, 20, 21). The Arabic version of FCOV-19S had an acceptable Cronbach alpha of 0.88 (22). The questionnaire was evaluated using a 5-point Likert scale ranging from 1 = “strongly disagree,” to 5 = “strongly agree.” The total score is calculated by adding all item scores to the 7 items, ranging from 7 to 35, wherein higher scores indicate greater fear of COVID-19.

Third, a short-form health literacy questionnaire (HLS-SF12) was used to measure the level of health literacy (20). HLS-SF12 was based on the original and comprehensive 47-item European Health Literacy Questionnaire (HLS-EU-Q47) developed by Sørensen et al. The HLS-SF12 consists of 12 items and is scored on a 4-point Likert Scale such as “1 = very difficult,” “2 = difficult,” “3 = easy,” and “4 = very easy.” An overall total score is computed by adding all item scores that represented the overall health literacy for each participant. Higher scores indicate better health literacy (range 0 to 50 points: 0 represented the lowest HL and 50 the highest). The HLS-SF12 has also shown satisfactory psychometric properties in different Asian countries (Cronbach's alpha = 0.85) (23).

### Data Analysis

Data analysis was done using SPSS version 23 (IBM, Chicago, IL, USA). All continuous variables were presented as mean ± SD while categorical variables were presented as frequencies (N) and percentages (%). Independent *t*-tests and ANOVA were used to determine the differences between fear of COVID-19 and their demographic characteristics. Pearson correlation was used to determine the association between the levels of fear of COVID-19 and health literacy. Multinomial logistic regression analysis was performed to identify predictors associated with fear of COVID-19 of the participants. A *p*-value was considered significant at *p* < 0.05.

## Results

The demographic characteristics of the participants are presented in Table 1. Of the 848 participants in the study, 56.8% were in the age group of 25–34, while 25.9% were 18–24 years old, and 12.5% in 35–44 years old. There were 461 male (54.4%) and 387 (45.6%) female participants. More than half of the participants were married (*N* = 472, 55.7%) and Saudi nationals (*N* = 488, 57.5%). Forty-two percent of the participants were employed and 33.8% were students. Among the participants, 57.1% had a bachelor's degree, 13.8% completed a master's or doctoral degree, and 14% had at least less than high school. In terms of family income, 70.6% of the participants had a family income of <10,000 SR (Saudi Riyal, SR 1 = USD.27). The analysis shows that the above-average total mean score for the FCoV-19S was FCoV-19: mean ± SD = 19.60 ± 7.33 (range 7–35) among the participants. With regards to the level of health literacy, the analysis showed that the participants had above average or marginal functional health literacy (HL index: mean ± SD = 27.57 ± 11.05) (range 0–50).

**Table 1:** Demographic characteristics of the participants.

**Variable**	**Count (*N* = 848)**	**%**
**Age**		
18–24	220	25.9
25–34	482	56.8
35–44	106	12.5
45 and above	40	4.7
**Gender**		
Male	461	54.4
Female	387	45.6
**Marital status**		
Single	376	44.3
Married	472	55.7
**Employment status**		
Employed	363	42.8
Student	287	33.8
Unemployment	198	23.3
**Nationality**		
Saudi	488	57.5
Non-Saudi	360	42.5
**Educational attainment**		
No formal education	62	7.3
Primary	26	3.1
Secondary	35	4.1
Diploma	124	14.6
Bachelors	484	57.1
Graduate	117	13.8
**Monthly income**		
<10,000 SR	599	70.6
10,000 or more	249	29.4
**FCoV-19, mean** **±SD**	19.60 (7.33)	
**HL index, mean** **±SD**	27.57 (11.05)	

Table 2 shows the difference between the levels of FCoV-19S of the participants. The analysis found that there is a significant difference in scores of fear of COVID-19 Scale in terms of age (*F* = 2.442, *p* = 0.050), representing that those aged 45 and above had higher mean fear scores than the younger participants. The score of fear of COVID-19 also significantly varied by the categories of employment and educational attainment. The results show that students (*M* = 20.37, SD = 7.31) and unemployed (*M* = 20.08, SD = 7.07) participants had a higher mean score on the FCOV-19S than employed participants (*M* = 18.80, SD = 7.48). The analysis of variance showed a significantly higher mean scale score on the FCOV-19S (*F* = 4.704, *p* = 0.001) in participants who had graduated (*M* = 20.76, SD = 7.20) and had a bachelor's degree (*M* = 20.22, SD = 7.07) than those who held primary education (*M* = 17.73, SD = 7.66) and no formal education (*M* = 16.75, SD = 7.62). The Pearson's correlation coefficients results showed a positive correlation between Fear of COVID-19 and the level of health literacy of the participants (*r* = 0.23, *p* = 0.011) (data not shown).

**Table 2:** Participants' scores on the fear of Corona Virus Disease—2019 (COVID-19) scale (FCoV-19S).

**Variable**	**Count (*N* = 848)**	**Statistical test**	***P-*value**
**Age**			
18–24	20.22 (6.50)	*F* = 2.442	**0.050**
25–34	19.49 (7.62)		
35–44	18.15 (7.54)		
45 and above	20.42 (7.33)		
**Gender**		*t* = −0.107	0.915
Male	19.57 (7.23)		
Female	19.63 (7.46)		
**Marital status**		*t* = 0.392	0.693
Single	19.51 (7.21)		
Married	19.71 (7.48)		
**Employment status**		*F* = 3.887	**0.021**
Student	20.37 (7.31)		
Unemployment	20.08 (7.07)		
Employed	18.80 (7.48)		
**Nationality**		*t* = 0.268	0.788
Saudi	19.66 (7.22)		
Non-Saudi	19.52 (7.49)		
**Educational attainment**		*F* = 4.704	**0.001**
No formal education	16.75 (7.62)		
Primary	17.73 (7.66)		
Secondary	18.97 (7.67)		
Diploma	18.08 (7.61)		
Bachelors	20.22 (7.07)		
Graduate	20.76 (7.20)		
**Monthly income**		*t* = −0.130	0.897
<10,000 SR	19.65 (7.35)		
10,000 or more	19.58 (7.33)		

*Bold values mean significant at P < 0.05*.

Multiple logistic regression was performed to examine the predictors of fear of Covid-19 of the participants. Of the eight variables entered in the analysis, 5 variables emerged as a significant predictor in the level of fear of COVID-19. Table 3 presented the details of the results. The analysis revealed an increased level of fear of COVID-19 was associated with older age (OR = 0.63; 95% CI 0.29–1.35, *p* < 0.020), being married (OR 1.40, 95% CI 1.09–1.44, *P* = 0.044), being unemployed (OR = 0.76; 95% CI 0.10–1.41, *p* = 0.023), with no formal education (OR = 0.21; 95% CI 0.31–0.16, *p* = 0.001) and low level of health literacy (OR = 0.88; 95% CI 0.63–1.12, *p*-value = 0.025).

**Table 3:** Predictors of the fear of COVID-19 identified by multivariable logistic regression analysis.

**Variable**	**Odd ratio**	**SE**	**95% CI**	***P*-value**
**Age**				
18–24	Reference			
25–34	1.15	0.39	0.53–2.49	**0.020**
35–44	0.91	0.35	0.45–1.85	
45 and above	0.63	0.38	0.29–1.35	
**Gender** (ref: male)	0.90	0.14	0.67–1.20	0.486
**Marital status** (ref: Single)	1.40	0.16	1.09–1.44	**0.044**
**Employment status** (ref: employed)	0.76	0.33	0.10–1.41	**0.023**
**Nationality** (ref: Saudi)	1.09	0.14	0.82–1.44	0.537
**Educational attainment**				**0.001**
No formal education	Reference			
Primary	0.21	0.34	0.31–0.16	
Secondary	0.37	0.45	0.37–0.15	
Diploma	0.47	0.39	0.47–0.21	
Bachelors	0.53	0.27	0.53–0.31	
Graduate	0.88	0.25	0.88–0.56	
**Monthly income** (ref: <10,000 SR)	1.01	0.18	0.70–1.45	0.953
**Health literacy index (ref: low health literacy)**	0.88	0.14	0.63–1.12	**0.025**

*Bold values mean significant at P < 0.05*.

## Discussion

This study investigated the level of fear of COVID-19 among the general population in Saudi Arabia and its association with their level of health literacy. The study highlights the level of fear of COVID-19 measured in the present study (*M* = 19.60; SD: 7.30), which was above the midpoint. The level of fear of COVID-19 among the general population in Saudi Arabia has been comparable to Turkey (*M* = 19.44), Japan (*M* = 18.71), Belarus (*M* = 16.6), and Russia (*M* = 17.4) (24–27). This shows the feeling of apprehension or fear of being infected among the general population in Saudi Arabia. In addition, the consequence of quarantine, lockdown, and other precautionary measures increases the level of stress, irritability, and insomnia among the participants. Recent studies show that restrictive public health measures and the effect of a pandemic such as COVID-19 impact individual mental well-being (7, 28, 29). Given the obtained overall mean for the fear of COVID-19 measure in the present study, it might be valuable for clinicians and health policymakers to consider developing timely public health strategies to mitigate the public concerns and fears related to COVID-19. Furthermore, the level of fear of COVID-19 found in the present study may cause negative lifestyle and behavior changes just to alleviate the feeling of fear.

This study also highlights the level of health literacy among the participants. The marginal level of health literacy found among the participants is comparable to studies in Germany and Australia (30, 31). The results of our study are parallel to the results of a nationwide study in Saudi Arabia, in which more than half of the participants had above the average or marginal level of health literacy (32). However, individuals who had low health literacy experienced difficulty in obtaining, understanding, and applying health information (33). During a disease outbreak, this factor may help in the effectiveness of public health measures to contain the virus. Because COVID-19 is novel, individuals must understand health information such as mode of transmission of the virus, signs and symptoms, and application of protective measures. Therefore, health policymakers, government, and healthcare providers might provide programs to improve the health literacy of the general population to minimize the consequence of the COVID-19 virus. For example, detailed information about COVID-19 prevention such as self-protective behaviors including proper washing of hands, wearing of a mask, and nutrition. Health literacy empowers people to make informed health decisions and to practice healthy and protective behaviors in the time of the coronavirus and COVID-19 pandemic (34–36). Therefore, enhancing individuals' health literacy skills is considered a strategic approach to reducing fear during this pandemic. An interdisciplinary approach is a key element for both information providers and receivers to have adequate health information that they can use to access, analyze, and apply during the COVID-19 pandemic.

In this study, we showed that there was a significant association between health literacy and fear of COVID-19 of the participants. Our findings were parallel to a previous study in Vietnam that found a significant association of fear of COVID-19 among medical students with health literacy (18). On the contrary, a previous study conducted in Pakistan in which they examine the impact of health literacy on fear of COVID-19 among university students shows that the level of health literacy did not predict their fear of COVID-19 (37). Given the variations in these findings, we call for more studies to provide a better understanding regarding the association of health literacy and fear of COVID-19. The novelty of the COVID-19 virus and the paucity of studies using specific health literacy measures do not yet allow the synthesis of the evidence on health literacy and COVID-19 management. Although there are inconsistencies, improving the health literacy of patients has been found to be an effective response in management and health outcomes (38). Health literacy could be useful and have an important role in this fight against the COVID-19 pandemic.

Another important finding was that a higher level of fear of COVID-19 scores was associated with older age, being married, being unemployed, with no formal education, and a low level of health literacy. This finding is similar to previous studies in Vietnam (18, 39). A study carried out in Vietnam among patients in the outpatient departments found that higher fear of COVID-19 scores had lower health literacy scores (39). The findings of the study indicate that an individual's level of health literacy has an impact on fear of COVID-19. Therefore, health education interventions are needed to be developed and improve the level of health literacy for communities particularly those with fears of COVID-19. Another approach that can be utilized is the use of mass media (e.g., health-mobile application) to provide accessible and reliable health information during the pandemic which can enhance patients' health literacy.

The present study acknowledges some limitations of the study. First, the data were derived online which we cannot assess the level of anxiety or depression of the participants. Moreover, the limited nature of the tools used in this study is based on symptoms and not on diagnosis. Second, the descriptive correlational design of the study could not determine causality. However, this study offers vital information to better understand the impact of the COVID-19 pandemic and that can be used as a baseline finding compared for future studies and with other populations.

In conclusion, the study shows the above-average level of fear of COVID-19 of the general public in Saudi Arabia and its association with a low level of functional health literacy. Notably, the findings show that age, employment status, educational attainment, and health literacy emerged as predictors and significant factors of fear of COVID-19. Timely and comprehensive health interventions should be promoted to enhance the level of health literacy of the community especially those individuals who were older, unemployed, and with no formal education. Moreover, public health interventions are suggested to reduce the level of fear of COVID-19 in the community which further protects against the transmission of the virus.

## Data Availability Statement

The data set used is locked and stored in the College of Nursing at Princess Nourah Bint Abdulrahman University and can be obtained from the author on reasonable request.

## Ethics Statement

The studies involving human participants were reviewed and approved by the Institutional Review Board, General Directorate of Health Affairs in Madinah (IRB no: 567, H-03-M-084).

## Author Contributions

All authors contributed to data analysis, interpretation of results, writing the manuscript, and agree to be accountable for all aspects of the work.

## Conflict of Interest

The authors declare that the research was conducted in the absence of any commercial or financial relationships that could be construed as a potential conflict of interest.

## Publisher's Note

All claims expressed in this article are solely those of the authors and do not necessarily represent those of their affiliated organizations, or those of the publisher, the editors and the reviewers. Any product that may be evaluated in this article, or claim that may be made by its manufacturer, is not guaranteed or endorsed by the publisher.
